# Impact of changing consumer lifestyles on the emergence/reemergence of foodborne pathogens.

**DOI:** 10.3201/eid0304.970409

**Published:** 1997

**Authors:** J E Collins

**Affiliations:** American Meat Institute, Arlington, Virginia 22209, USA. ami@interramp.com

## Abstract

Foodborne illness of microbial origin is the most serious food safety problem in the United States. The Centers for Disease Control and Prevention reports that 79% of outbreaks between 1987 and 1992 were bacterial; improper holding temperature and poor personal hygiene of food handlers contributed most to disease incidence. Some microbes have demonstrated resistance to standard methods of preparation and storage of foods. Nonetheless, food safety and public health officials attribute a rise in incidence of foodborne illness to changes in demographics and consumer lifestyles that affect the way food is prepared and stored. Food editors report that fewer than 50% of consumers are concerned about food safety. An American Meat Institute (1996) study details lifestyle changes affecting food behavior, including an increasing number of women in the workforce, limited commitment to food preparation, and a greater number of single heads of households. Consumers appear to be more interested in convenience and saving time than in proper food handling and preparation.


### Impact of Changing Consumer Lifestyles on the Emergence/Reemergence of Foodborne Pathogens

**Janet E. Collins**
American Meat Institute, Arlington, Virginia, USA

Foodborne illness of microbial origin is the most serious food safety problem in the United States. The Centers for Disease Control and Prevention reports that 79% of outbreaks between 1987 and 1992 were bacterial; improper holding temperature and poor personal hygiene of food handlers contributed most to disease incidence. Some microbes have demonstrated resistance to standard methods of preparation and storage of foods. Nonetheless, food safety and public health officials attribute a rise in incidence of foodborne illness to changes in demographics and consumer lifestyles that affect the way food is prepared and stored. Food editors report that fewer than 50% of consumers are concerned about food safety. An American Meat Institute (1996) study details lifestyle changes affecting food behavior, including an increasing number of women in the workforce, limited commitment to food preparation, and a greater number of single heads of households. Consumers appear to be more interested in convenience and saving time than in proper food handling and preparation.

Reporting of foodborne and waterborne diseases in the United States began more than 50 years ago (1). At that time, state and territorial health offices were concerned about the levels of morbidity and mortality caused by typhoid fever and infantile diarrhea; cases were to be investigated and reported. The underlying purpose of reporting was to obtain information regarding the role of food, milk, and water in outbreaks of intestinal illness to provide a basis for public health action.

In 1923, the Public Health Service began publishing summaries of outbreaks of gastrointestinal illness attributed to milk; in 1938, it added summaries of outbreaks due to any foods. In 1966, the present system of surveillance of foodborne and waterborne diseases began to incorporate into an annual summary all reports of enteric disease outbreaks attributed to microbial or chemical contamination of food or water. Comprehensive surveillance should result in greater awareness of the most important food-protection methods.

Address for correspondence: Janet E. Collins, American Meat Institute 1700 N. Moore Street, Suite 1600, Arlington, VA 22209 USA; fax: 703-527-0938; e-mail: ami@interramp.com.

Between 1983 and 1987, the etiologic agent in foodborne disease outbreaks was not determined in 62% of the outbreaks (2); between 1988 and 1992, the foodborne disease was of unknown etiology in 59% of the outbreaks (1). Bacterial pathogens caused the largest percentage of outbreaks (79%) when etiology was known—Salmonella caused 69% of bacterial outbreaks. For each year from 1983 through 1992, the most commonly reported food preparation practice that contributed to foodborne disease concerned improper holding or storage temperatures. The second most common practice was poor personal hygiene of the food handler. Food from unsafe sources was the least commonly reported factor in each of the 10 years of reporting. It is now time to examine food handling and determine how to reverse the trend.

Foodborne disease surveillance has traditionally served three purposes. The first is disease prevention and control. Prevention and control measures include early identification and removal of contaminated products from the commercial market and correction of faulty food-preparation practices in both food-service establishments and the home. Surveillance also provides knowledge of disease causation. The responsible pathogen is not identified in more than half of the foodborne disease outbreaks for various reasons, including late initiation of laboratory investigation, inability of available technology to identify the pathogen, and lack of identification of the pathogen with a particular food. Finally, surveillance assists in administrative guidance. Information enables assessment of trends in prevalence of outbreaks caused by specific etiologic agents and in vehicles of transmission. This information assists in identifying common errors in food handling. In July 1995, the Centers for Disease Control and Prevention (CDC), Food and Drug Administration (FDA), and Food Safety and Inspection Service (FSIS) began a comprehensive effort to track major bacterial pathogens that cause foodborne illnesses (3). CDC provides the overall management and coordination with state health departments in the five survey sites of the FSIS/CDC/FDA Sentinel Site Study. The program actively seeks out specific cases of foodborne illness to identify whether a food was of concern and to better establish frequency and source of foodborne disease outbreaks and cases. CDC will use the data to identify emerging foodborne pathogens and monitor incidence of foodborne illness; FSIS will use the data to evaluate the effectiveness of new food-safety programs and regulations to reduce foodborne pathogens in meat and poultry; FDA will use the data to evaluate its efforts to reduce foodborne pathogens in seafood, dairy products, fruits, and vegetables.

According to a recent report to Congressional committees (4), experts believe that the risk for foodborne illness is increasing. The food supply is changing in ways that can promote foodborne illness, and there are no comprehensive data to explain at what point pathogens are introduced into food. Further, because of demographic changes, more people are at a greater risk of contracting a foodborne illness.

According to Ollinger-Snyder and Matthews (5), changes in agricultural practices, a growing population susceptible to infectious diseases, lifestyle changes, the emergence of new foodborne pathogens, and the high turnover rate reported for workers in the food-service industry indicate that new approaches are needed to allay consumers' fears and to prevent the spread of foodborne disease in the United States. They recommend implementation of Hazard Analysis and Critical Control Points (HACCP) systems and certification of food-service managers.

The food processing industries are developing and implementing HACCP systems; the meat and poultry industries are mandated to do so beginning in January 1998 (6). Hazard analysis has been defined as the identification of sensitive ingredients, critical processing points, and human factors that affect product safety. Critical control points have been described as processing determinants whose loss of control would result in an unacceptable food-safety risk.

Most contend that the HACCP system approach must be implemented at each stage of the farm-to-family continuum. Where are the critical control points and the HACCP system development in the home, food-service or retail establishments, or the car when food is carried from one location to another? The consumer is a complex and critical control point in the process.

Take the case of the barbecued chicken served to 260 guests at an outdoor barbecue in 1983. Guests were served chicken that was parboiled in the morning by one set of cooks and then placed in a large container and refrigerated. The evening cooks assumed the chicken had been adequately cooked, so they basted it in barbecue sauce and warmed it over the fire. Some 71% of the guests got sick from the chicken that was insufficiently cooked and improperly held (5). What of the infected bakery worker who stirred a vat full of buttercream frosting with a bare hand and arm? Some 5,000 cases of viral gastroenteritis were caused by the infected worker who claimed he had washed his hands. Other more recent outbreaks (7) appear in Table 1.

Recent data (1) indicate that 80% of reported foodborne illness outbreaks occur outside the home. Even though illnesses would be expected to be reported more often when they occur as a result of eating in restaurants, the numbers are large. National standards for restaurant safety are contained in the Food Code (8). FDA has the legal authority to impose the standards on state and local jurisdictions. The Food Code, which is updated every 2 years, includes temperatures for cooking, cooling, refrigeration, reheating, and holding food in food-service establishments. County or city employees are generally charged with responsibility for inspecting restaurants; each state or locality has its own laws governing restaurant safety.

Table 1. Foodborne illness reports from restaurants, 1996

| Date | Description | Cause |
|---|---|---|
| 6/96 | Salmonella-contaminated food, 38 cases | Employees did not wash hands before handling food |
| 9/95 | *Escherichia coli* O157:H7 "beef," 11 cases | Raw food cross-contaminated other |
| 8/95 | *Salmonella* Newport "chicken," >850 cases | Raw meat on cutting board with vegetables |
| 1/95 | Hepatitis A, contaminated food, 95 cases | Human fecal matter from handling—handwashing |
| 8/94 | Salmonella, hollandaise sauce, 56 cases | Holding temperature too low for 9 hours |
| 1/93 | *Clostridium botulinum*, canned cheese sauce, 7 cases, 1 death | Unrefrigerated storage of opened container |

Source: Center for Science in the Public Interest, 1996

Food service outside the home is big business, with sales of more than $300 billion (9) and nearly 10 million employees. The restaurant industry's share of the food dollar is 43%, and the typical consumer more than 8 years of age had more than four meals per week away from home in 1996. Given those statistics, it is clear that food-service establishments play a critical role in food safety. The Center for Science in the Public Interest (7) conducted a survey of 45 agencies across the country to determine if state and local agencies were enforcing 12 key food-safety standards in the FDA Food Code. The standards chosen for the study affect consumer health and safety and include such areas as food cooking and refrigeration temperatures, frequency of inspections, and consumer warnings for raw foods. Not one of the 45 agencies surveyed was following all of the Food Code recommendations.

In the survey, only 13% of agencies enforced the Food Code and recommended cooking temperatures for pork, eggs, fish, and poultry; only 64% of agencies required hamburgers to be cooked to 155°F. Recommendations for cooling cooked food were followed by only 20% of the agencies, and only 11% required refrigeration of food at FDA-recommended temperatures.

Every restaurant can take steps to ensure the safety of the food it prepares and serves to its customers. Continuous employee training and institution of HACCP-type systems should assist restaurants and other food-service institutions in improving their food-safety records. Programs available through the national restaurant trade organization could assist even the smallest establishments in achieving food-safety goals.

For more than 25 years, the Food Marketing Institute (10) has surveyed consumers about their changing needs and priorities in food attitudes and behavior. The 1996 trends report has an expanded focus on the primary grocery store or supermarket, including questions to help retailers learn more about take-out foods (Table 2). In 1996, nearly 40% of the 2,000 shoppers surveyed purchased fresh deli items from their primary supermarket at least once per week, and more than 10% reported purchasing ready-to-eat take-out foods as frequently. Three-fourths of these shoppers purchased food from the deli at least once per month, and half bought take-out food from the supermarket as often.

According to the survey, fast-food restaurants dominate (48%) all food outlets as the primary source of take-out food; only 12%

Table 2. Sources of take-out food (%)

| Source | 86 | 87 | 88 | 89 | 90 | 91 | 92 | 93 | 94 | 95 | 96 |
|---|---|---|---|---|---|---|---|---|---|---|---|
| Fast-food restaurant | 43 | 44 | 41 | 41 | 46 | 51 | 55 | 46 | 46 | 41 | 48 |
| Restaurant | 38 | 33 | 38 | 33 | 27 | 23 | 24 | 27 | 25 | 22 | 25 |
| Supermarket | 10 | 9 | 11 | 12 | 14 | 14 | 12 | 15 | 15 | 17 | 12 |
| Deli/pizza parlor/bagel shop/coffee shop/donut shop | * | * | * | * | * | * | * | * | * | 8 | 4 |
| Gourmet or specialty shop | * | * | * | * | * | * | * | * | * | * | 3 |
| Convenience store | * | * | * | * | 2 | 2 | 2 | 2 | 2 | 2 | 1 |
| Some other place | 2 | 7 | 3 | 6 | 2 | 1 | * | 1 | 1 | 1 | 2 |
| It varies | 1 | 1 | 1 | 3 | 4 | 5 | 3 | 5 | 3 | 2 | 0 |
| Don't eat out | * | * | * | 7 | 6 | 4 | 4 | 4 | 4 | 3 | 2 |
| Not sure | 3 | 3 | 4 | 1 | * | 1 | 1 | 1 | 2 | 3 | 2 |

*Data not collected for this year.
Source: Food Marketing Institute, 1996

purchase take-out foods from the supermarket. A recent article in Food Processing Magazine (11) states, "Somewhere on their way to the supermarket, consumers have been getting lost." Home-meal replacement, ready-made meals approximating what Mom used to make, have begun to rapidly compete for the food dollars of time-pressed consumers. According to Hollingsworth (12), consumers are eating more meals at home, but they are not cooking more. Consumers want to get food in a take-out location and go home to eat it (Figure).

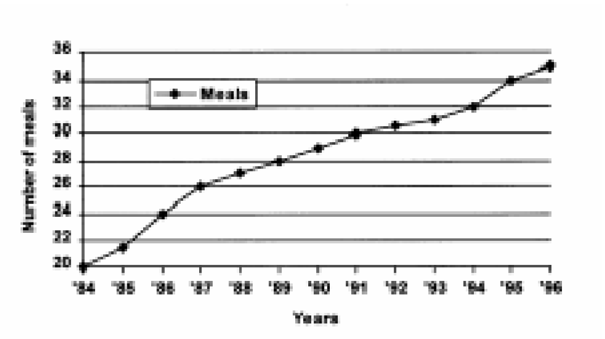

Figure. Annual meals (including snacks) purchased at commercial restaurants per person and consumed at home.

These take-out or eat-at-home foods have built-in food-safety hazards. Consumers are time-pressed, and they are buying these foods. Are they treating them as perishable? The U.S. Department of Agriculture (13) has expressed concerns about these foods; they say that take-out foods need to be handled with care. Hot foods need to be picked up or received hot and eaten within 2 hours. If eaten later, hot foods should be divided into shallow containers, covered loosely, and refrigerated immediately.

Are consumers ready for all of this food handling? Most consumers are confident that the food they purchase is safe to eat (10). Spoilage of foods is considered the greatest threat to food safety by the largest group (49%) of respondents. They count on freshness and expiration dates (22%) and increasingly see bacteria and contamination as threats (17%). It is interesting to note, however, that between 1992 and 1996 these shoppers were less likely (15% vs. 7%) to see spoilage as a threat; similarly, processing and preparation of foods was less an issue in 1996 than in 1992 (8% vs. 10%).

Consumers are concerned about handling of foods by other shoppers and by supermarket employees. Consumers rely increasingly on food stores (16%), manufacturers (21%), government (21%), and themselves (25%) for food-safety protection. Consumers apparently are willing to share responsibility for food safety with others, but they want to know that steps are taken during the processing and distribution of foods to reduce the likelihood of pathogen or other bacterial contamination.

According to Technomics (14), these supermarket issues noted in the Food Market Institute trends data (10) will be shared with food-service operators as the share of consumer food expenditures changes from 51% vs. 49%, 48% vs. 52%, and 45% vs. 55% (projected) for retail expenditures versus food-service expenditures in 1991, 1996, and 2001, respectively.

The number of households earning more than $75,000 annually continues to grow, and these households exhibit the highest levels of spending on food service. Consumer demands are changing the way that food-service operators and suppliers of food services must react. The area of convenience, highly prized by consumers today, has profound implications for food. Consumers want fast service with easy-to-eat foods and no stress, which means a far greater emphasis on portable foods.

Technology has conditioned us to demand and receive near-immediate satisfaction. There will be even greater emphasis on faster service, meaning more emphasis on convenient food formats to expedite preparation. Packaging and storage will greatly affect product quality and safety. According to Technomics (14) packaging will need to be temperature-tolerant and breathable. Preparation and processing technologies will need to have greater ability to rapidly cool and chill. And then there is the food-safety concern associated with dispensing equipment. Food will be required to have an extended shelf life. The safety factors associated with these new formats will also change.

Consumers want easy access to portable foods. Accessibility to variety in food options translates to a proliferation in nontraditional locations. These smaller sites may include back-of-house preparation facilities. This easy access to smaller operations also suggests a need for more of such operations and more variety in menu options. While to the consumer this may translate to upscale menus with indulgence foods such as new and different bakery items, microbrewery beverages, and gourmet coffees, to the food-service operator it may mean greater cross-contamination with cream fillings, unpasteurized fermented drinks, and spoiled milk. New menu options create new challenges for service and for safety.

According to Steve Harrison, brewmaster of the Sierra Nevada Brewing Company (Chico, CA), “The concept that a beer will automatically go bad in ‘X’ number of days is a very untrue one.” Consumers do not know that. What is “skunky beer”? Starting in late 1996, Anheuser-Busch began a freshness strategy in their advertising. Other large brewers are catching on, so freshness is associated with quality and safety. Imagine freshness dating, “born-on dating,” as a quality parameter in brewing.

Consumers’ increased emphasis on food-safety issues directly affects food service. The perceived healthfulness and quality of foods affects food sales; the increasing considerations of cleanliness as healthfulness and quality as safety become even greater shared responsibilities as food-service operators take over the roles historically associated with home kitchens. “On-the-spot exhibition” cooking is of increasing interest to today’s consumers.

In June 1996, the Food Marketing Institute (15) published a review of foodborne illness. They note that the organisms that cause foodborne illnesses are found throughout nature and that mishandling and poor refrigeration are responsible for most contamination. The most common causes are cross-contamination of cooked foods with raw foods, contaminated utensils or serving plates, poor hygiene of food handlers, and time or temperature abuse.

Agreement is widespread that the most serious food-safety problem is foodborne illness of microbial origin (Table 3). Foodborne pathogens include a wide array of microorganisms, which have various physiologic effects on people, ranging from mild to severe, and are associated with a wide array of foods. Cross-contamination and association of foods within mixed dishes complicate environmental control. Further, some of the microbes have evolved and become more resistant to food preparation and storage techniques. Several industry and government publications (1,2,8,15,16) summarize biologic hazards associated with foodborne illness.

Mishandling can occur at any point in the food chain—in processing, at supermarkets or restaurants, or in homes. Many food manufacturers and retailers have HACCP plans in place, and over the next few years that number will increase. Consumers, however, must assume responsibility for the safety of food in the home. Proper preparation and sanitation methods are key to preventing foodborne illness in the home as in other areas of food handling. The messages for each of the segments of the food chain are the same—keep it clean (e.g., wash your hands) and control the temperatures (keep hot things hot and cold things cold) (Table 4).

For the food-service industry, a number of programs have been developed to educate food

Table 3. Sources of reported outbreaks with confirmed causes (%)

| | Restaurant | | Other Known Place | | |
|---|---|---|---|---|---|
| | 1983-87 | 1988-92 | 1983-87 | 1988-92 | 1996 |
| *Salmonella* | 50 | 60 | 46 | 58 | 30 |
| *Escherichia coli* | <1 | <1 | 2 | 1 | 5 |
| Hepatitis A virus | 6 | 7 | 3 | 4 | |
| *Staphylococcus aureus* | 2 | 3 | 10 | 5 | |
| *Campylobacter* | 1 | 2 | 6 | 3 | 45 |
| *Shigella* | 8 | 2 | 6 | 2 | 17 |

Sources: Centers for Disease Control and Prevention and U.S. Department of Agriculture for 1996, the 1996 Sentinel Site Study

Table 4. Pathogen control in foods to reduce foodborne illness

| Pathogen | Control mechanism |
|---|---|
| *Campylobacter* | Heat foods ≥ 140°F<br>Proper handling |
| *Salmonella* | Rapid chilling <40°F<br>Hot storage >140°F<br>Cooking >165°F |
| *Escherichia coli* O157:H7 | Heat foods >155°F<br>Avoid cross-contamination |
| *Staphylococcus aureus* | Rapid cooling <40°F<br>Personal hygiene |
| *Clostridium botulinum* | Boil food 10-15 minutes |
| *Clostridium perfringens* | Refrigerate <40°F<br>Proper handling |
| *Listeria* | Pasteurization of milk<br>Adequate cooking |

Source: Food Marketing Institute, 1996

handlers about food-related and personal behaviors that affect the safety of foods. For example, the Food Marketing Institute (17) has a Food Protection Certification Program for supermarket personnel to learn about the FDA Food Code requirements regarding food handling and hygiene. Similarly, the National Restaurant Association has developed a food-safety program called Serve Safe, intended to educate foodservice workers about safe food handling.

Who or what teaches the average consumer about food safety? Common sense? Family? Health and fitness magazines? In May 1996, the Food Marketing Institute (17) conducted a series of consumer focus groups to establish the importance of food safety to consumers and to identify barriers to consumers' safe food purchase, handling, and preparation. They report that how consumers manage food safety reflects years of conditioning, observation, and reinforcement from mothers and grandmothers. In some cases, the more often consumers shop, the less concerned they seem to be about food safety when it comes to shopping, storage, and handling. Consumers link safety to fresh food, and they assume that when they shop more often, they purchase food in smaller quantities and food safety is less an issue. Respondents in the study also tended to think that cooked food was generally "safer" than raw food. For example, they believed that recontamination of unrefrigerated food was less a problem with cooked than with raw food.

Some safe food practices are observed for convenience, esthetics, or taste rather than for food safety. Thawing meat is messy; covering food prevents it from drying out; separating foods in the refrigerator is tidier. These kinds of behavior improve safety, but consumers may not understand the food-safety implications.

Overall, the consumers in the Food Marketing Institute study (17) find food-safety messages generally are "common sense," "basic," "practical," and "believable." Messages about such subjects as the order in which to choose foods in the supermarket, sell-by dates, storage and freezing of products, ways of keeping hot foods hot and cold foods cold are not considered too elementary. They also believe that storage times for food safety do not apply equally across food groups; they do not understand hazards from vegetables or fruits. Barriers to safe food-handling behavior in this study included historical (and cultural) practices, feeling of invulnerability, taste preferences, timing and planning, and space and convenience.

A 1992 survey conducted at Cornell University and designed to assess consumer food-safety awareness documented a substantial lack of knowledge about safe home food preparation practices. Seventy-five percent of those surveyed knew that Salmonella is associated with meat, poultry, and eggs, but only 65% would refrigerate a roasted chicken breast immediately; 29% would leave it on the kitchen counter until it reached room temperature. Further, 18% said they would not be concerned or were not sure about the safety of cooked meat left unrefrigerated for more than 4 hours; 14% said the same for cooked poultry.

In April 1996, the American Meat Institute (16) commissioned a study of 1,000 adults in the United States. Compared with 98% of respondents in the study who know that harmful bacteria can be present on meat and poultry products, only 74% made the link to dairy products and eggs; two in five respondents (43%) recognized that fruits and vegetables may contain harmful bacteria. These conclusions could be drawn for consumers who responded to the American Meat Institute (1996) questions. While the U.S. population is growing (up 10% since 1980), households are becoming smaller. In the 1980s, the number of households grew 17%, while the average household size decreased from 2.8 to 2.6 persons. This shift in family size and the increase in single heads of households has resulted in increased stress in the family with less time for shopping and food preparation. In addition, more women are in the workforce. Today, 70% of women ages 25 to 44 years are in the workforce; 75% work full time. Therefore, no adult is likely to be in the home for 70% of American households, and many children are preparing food for themselves. Finally, consumers spend less time on food preparation. More than 85% of employed women shop and cook, but most spend less than 30 minutes preparing every meal and 20% spend less than 15 minutes. Consumers are using convenience foods and quick methods of food preparation, including partially cooked foods that may require special handling.

The study results provided further documentation that the risk for foodborne illness is increasing, largely because of societal changes that affect the way consumers purchase and prepare food. Contributing to this are changes in the family structure, more women in the workforce, and less available time for food preparation. Consumers in this study were not able to correctly separate home preparation issues from food service, nor did they know correct cooking temperatures to use in their own homes.

The ways in which consumers spread microorganisms to one another and to themselves include more than just coughing and sneezing. Not washing hands before, during, and after handling foods clearly contributes to the spread of foodborne infections and intoxication. Hands can spread disease-causing microbes to foods from other foods and from infected persons.

In a comprehensive review of 91 scientific articles published after 1986, Bryan et al.(18) attempted to link hand washing and infections. They report that hand washing has become an integral component of the tradition and ritual of prevention practice for the spread of infection, but several factors confound the ability to establish the effectiveness of hand washing for reducing infectious disease. Hand-washing practices were shown to significantly reduce infections transmitted by the fecal-oral route and in situations of poor personal hygiene. Hand washing is clearly a critical step in reducing personal contamination of food and cross-contamination between foods. Hand washing is but one practice that could dramatically affect risk, if not incidence, of foodborne disease.

According to data provided by the American Society for Microbiology (19), people do not wash their hands as often as they think they do (Table 5). In telephone surveys, 94% of respondents claim they always wash up after using the rest room; however, researchers contend that almost one-third of people do not wash their hands after using the bathroom. Of the more than 7,000 people nationwide who participated in the study, 81% said they wash their hands before handling or eating food. However, most say they do not wash up after petting an animal (48%), coughing or sneezing (33%), or handling money (22%).

Table 5. American Society for Microbiology/Bayer hand-washing survey, 1996

| Behavior/Location | What they say[a] (%) | What they do[b] (%) |
| --- | --- | --- |
| Wash hands: | | |
| After using public restroom | 94 | 68 |
| Women | | 74 |
| Men | | 61 |
| New York (Penn Station) | | 60 |
| Chicago (Navy Pier) | | 78 |
| New Orleans (casino) | | 71 |
| San Francisco (Golden Gate Park) | | 69 |
| Atlanta (Braves game) | | 64 |
| Women | | 89 |
| Men | | 46 |

[a]1,004 adults; [b]6,330 adults
Source: American Society for Microbiology

In early 1997 (8), the U.S. Departments of Agriculture and Health and Human Services and the U.S. Environmental Protection Agency developed a program intended to coordinate a food-safety initiative among federal agencies, immediately after an announcement by U.S. President Clinton (January 1997) to promote an initiative designed to improve the safety of the nation's food supply. The president charged the federal agencies to work with consumers, producers, industry, states, tribes, universities, and the public to identify ways to improve food safety through government and private sector action, including public-private partnerships. The interagency response is a multifaceted program designed to include surveillance, coordination of activities within the various programs and agencies, risk assessment, research, inspections, and education. The underlying premise upon which this program was developed is that foodborne infections remain a major public health problem. Further, sources of food contamination are said to be almost as numerous and varied as the contaminants; bacteria and other infectious organisms are pervasive in the environment.

The current systems for protecting food in the United States include a broad range of government agencies and industries, many of which have been discussed in this paper. Responsibilities are shared among the U.S. Department of Agriculture (Food Safety and Inspection Service and Animal and Plant Health Inspection Service), the U.S. Department of Health and Human Services (FDA and CDC), and the U.S. Environmental Protection Agency. These responsibilities include oversight on the farm, in the processing facilities, during transportation and distribution (including food from foreign countries), and in food marketing channels including restaurants, supermarkets, and institutional food services (such as schools and hospitals).

Surveillance of foodborne illness outbreaks and their causes is a responsibility of FDA and

CDC. Education is shared among the agencies and is not the primary concern or responsibility of any one of the agencies. Pivotal to this new initiative is the element of education. Specifically, the program is intended to reinvigorate education of all those involved in food preparation, focusing on the use of safe practices. According to USDA et al. (8), educating people about steps they must take to prevent and control foodborne illness is a vital link in the food preparation chain. In spite of the education efforts of the government, both state and federal, consumer groups, and industry, which have occurred historically, foodborne illness occurs from a lack of knowledge of the risks involved at all stages of food preparation. Choices consumers make about how they handle food at home and about eating food that increases the risk for illness can have an important effect on foodborne disease incidence.

USDA et al. (8) will develop a program to improve consumer education; retail, food service, and institutional education; veterinary and producer education; and industry education in the transportation area. They propose developing an alliance among industry, consumer groups, and governmental agencies to mount a comprehensive food-safety awareness campaign for consumers. Highly focused messages and tactics for the general public and consumers at high risk will be developed. This thrust is in perfect harmony with the strategies and tactics proposed by the American Meat Institute (16) as an outcome to a series of studies and roundtable discussions held with medical doctors, dietitians, educators, and others. The ability of industry and consumer groups to work with the government in a program with common themes and elements is critical to the positive outcome of the effort. As one of the focus group members in the Food Marketing Institute (17) said, the more often the message is repeated, the more likely is the listener to hear it.

The broad-based approach to education, which includes data from surveillance and inspections, should provide the foundation for changes in consumer behavior. It is critical that consumers not only take responsibility for their actions regarding food safety, but that they also take seriously the learning that must occur for consumers of all ages to prevent contamination, cross-contamination, and mishandling of foods at home and in restaurants. Convenience, taste, and variety are welcome qualities in foods that we enjoy; safety in foods is critical to the public health and safety of consumers and to the government and businesses that support those consumers.

### References

1. Centers for Disease Control and Prevention. Surveillance for foodborne disease outbreaks, United States, 1988—1992. MMWR CDC Surveill Summ 1996;45:1-66.
2. Centers for Disease Control. Foodborne disease outbreaks, 5-year summary, 1983—1987. MMWR CDC Surveill Summ 1990;39:15-57.
3. Food Safety and Inspection Service. FSIS/CDC/FDA sentinel site study: the establishment and implementation of an active surveillance system for bacterial foodborne diseases in the United States. Washington (DC): USDA Report to Congress; 1997.
4. United States General Accounting Office. Food safety: information on foodborne illnesses. Report to Congressional Committees. Washington (DC): GAO/RCED-96-96; 1996.
5. Ollinger-Snyder P, Matthews ME. Food safety issues: press reports heighten consumer awareness of microbiological safety. Dairy, Food and Environmental Sanitation 1994;14:580.
6. FSIS Pathogen Reduction/HACCP. Washington (DC): Federal Register 1996 Jul 6.
7. Center for Science in the Public Interest. Dine at your own risk: the failure of local agencies to adopt and enforce national food safety standards for restaurants. Washington (DC): The Center; 1996.
8. United States Department of Agriculture, United States Department of Health and Human Services, United States Environmental Protection Agency. Food safety from farm to table: a new strategy for the 21$^{ST}$ century. Discussion Draft. Washington (DC): United States Department of Agriculture; 1997.
9. National Restaurant Association. In: 1997 Restaurant Industry Pocket Factbook. Washington (DC): The Association; 1997.
10. Food Marketing Institute. Trends in the United States: consumer attitudes and the supermarket. Washington (DC): The Institute; 1996.
11. Neff J. Will home meal replacement replace packaged foods? Food Processing 1996;57:35.
12. Hollingsworth P. The changing role of fast food. Food Technology 1997;51:24.
13. United States Department of Agriculture, Food Safety and Inspection Service. Take out foods—handle with care. The Food Safety Educator 1996;1:2.
14. Management summary update: critical strategic issues 1996-2001. Chicago: Technomics, Inc.; 1997.
15. Food Marketing Institute. Backgrounder: foodborne illness. Washington (DC): The Institute; 1996.
16. American Meat Institute. Putting the food-handling issue on the table: the pressing need for food safety education. Washington (DC): American Meat Institute and Food Marketing Institute; 1996.

17. Food Marketing Institute. Food safety: a qualitative analysis. Washington (DC): The Institute; 1996.
18. Bryan JCL, Chorine J, Larson EL. Handwashing: a ritual revisited. Infection and Control in Critical Care 1995;7:617-24.
19. American Society for Microbiology. Americans get caught dirty handed. Fact Sheets. Washington (DC): The Society; 1996.

